# Microbiome of esophageal endoscopic wash samples is associated with resident flora in the esophagus and incidence of cancer

**DOI:** 10.1038/s41598-024-67410-1

**Published:** 2024-08-22

**Authors:** Takuya Shijimaya, Tomomitsu Tahara, Jumpei Yamazaki, Sanshiro Kobayashi, Yasushi Matsumoto, Naohiro Nakamura, Yu Takahashi, Takashi Tomiyama, Toshiro Fukui, Tomoyuki Shibata, Makoto Naganuma

**Affiliations:** 1 Third Department of Internal Medicine, Kansai Medical University, 2-5-1 Shin-Machi, Hirakata, Osaka 573-1010 Japan; 2 Translational Research Unit, Faculty of Veterinary Medicine, Veterinary Teaching Hospital, Hokkaido University, Sapporo, Japan; 3 One Health Research Center, Hokkaido University, Sapporo, Japan; 4 Department of Gastroenterology, Fujita Health University School of Medicine, Toyoake, Japan

**Keywords:** Esophageal carcinoma, Microbiome, Esophageal endoscopic wash, 16S rRNA, Diagnosis, Cancer, Gastroenterology, Oncology

## Abstract

Change in mucosal microbiome is associated with various types of cancer in digestive tract. We hypothesized that microbial communities in the esophageal endoscopic wash fluids reflects resident flora in esophageal mucosa that is associated with esophageal carcinoma (EC) risk and/or directly correlates microbiome derived from EC tumor tissue. Studying microbial communities in esophageal endoscopic wash samples would be therefore useful to predict the incidence or risk of EC. We examined microbial communities of the endoscopic wash samples from 45 primary EC and 20 respective non-EC controls using 16S rRNA V3-V4 amplicon sequencing. The result was also compared with microbial communities in matched endoscopic biopsies from EC and non-cancerous esophageal mucosa. Compared with non-EC controls, 6 discriminative bacterial genera were detected in EC patients. Among them, relative abundance ratio of *Prevotella* and *Shuttlewarthia,* as well as decrease of genus *Prevotella* presented good prognostic performance to discriminate EC from controls (area under curve, 0.86, 0.82, respectively). Multivariate analysis showed occurrence of EC was an independent factor associated with decrease of this bacteria. Abundance of genus *Prevotella* in the esophageal endoscopic wash samples was significantly correlated with the abundance of this bacteria in the matched endoscopic biopsies from non-cancerous esophageal mucosa but not in the EC tissues. Our findings suggest that microbiome composition in the esophageal endoscopic wash samples reflects resident flora in the esophagus and significantly correlates with the incidence of EC.

## Introduction

Esophageal carcinoma (EC) is an aggressive malignancy, with an annual incidence of 600 000 cases around the world^1^. Squamous cell carcinoma (SCC) is the first histologic subtype of esophageal cancer in Eastern and Central Asia, accounting for about 80% of the total cases^2^, while esophageal adenocarcinoma accounts for more than half of esophageal cancers in western countries^3–5^ and its incident rate is also gradually increasing in Japan^6,7^. Given the poor prognosis of late-stage EC^3–5,8,9^, deeper knowledge of the pathogenesis that drives the transition from normal epithelium to EC may provide new therapeutic alternatives, improving diagnostic and prognostic tools to accurately refine the individual risk.

There is growing evidence linking abnormal changes in the gastrointestinal (GI) microbiota, with several GI^10–12^ and extra-intestinal diseases^13–16^, and, also, with various types of cancers^17,18^. One of the best described examples is *Fusobacterium* species in colorectal tumorigenesis^19,20^. *Fusobacterium nucleatum* (*F. nucleatum*) is detected at higher quantities in colorectal cancer and adenoma tissues^19,20^. The mechanistic role of *Fusobacterium* species in colonic tumorigenesis has been demonstrated by altering the host immune responses^20^ and inter-or intra-cell signaling^21^. *F. nucleatum* selectively recruits tumor-infiltrating myeloid cells, which can promote tumor progression^20^. *Fusobacterium* high human colorectal cancers also exhibit a pro-inflammatory signature characterized as increased NF-κB activation and expression of pro-inflammatory genes such as *PTGS2*, *IL1β*, *IL6*, *IL8*, and *TNF- α*^20^. It has also been reported that adhesin FadA, a virulence factor identified from *F. nucleatum* binds to E-cadherin, activates β-catenin signaling, promotes oncogenic responses^21^.

For another pathogenic microbiome, colibactin-producing *E*. *coli* is also thought to be associated with colorectal cancers inducing DNA double-strand breaks and gene mutations^22^.

Several cross-sectional studies have also suggested an association between the etiology of EC and microbial dysbiosis in the upper digestive tract^23,24^. Presence of *Fusobacterium*, well known colorectal cancer microbiome, is also reported to be associated with poor prognosis esophageal SCC^25^. Recent study reported the microbial composition in esophageal SCC using esophageal lavage samples^26^. Lavage samples derived from endoscopic wash fluid can potentially cover broader mucosal layer of targeted organ. It has been reported that investigating endoscopic wash fluid could represent an alternative for the detection of gastrointestinal cancers as noninvasive strategy^27^. We hypothesized that microbial communities in the esophageal endoscopic wash fluids reflects resident flora in esophageal mucosa that is associated with EC risk and/or directly correlates microbiome derived from EC tumor tissue. Studying microbial communities in esophageal endoscopic wash samples would be therefore useful to predict the incidence or risk of EC. To test this hypothesis, we conducted a case–control study to characterize the microbial communities in esophageal endoscopic wash samples from 45 primary esophageal carcinoma (EC) and 20 respective non-EC controls using 16S rRNA V3-V4 amplicon sequencing. We obtained endoscopic wash samples derived from entire area of esophagus.The result was also compared with microbial communities in matched endoscopic biopsies from EC and non-cancerous esophageal mucosa.

## Materials and methods

### Study population, sample collection

This study examined patients with 45 primary esophageal carcinoma (EC) and 20 respective non-EC controls attending the endoscopy Center of Fujita Health University and Kansai medical university. This study was approved by the Institutional Review Board of Fujita Health University (ID: HM18-094) and Kansai medical university (ID: 2,021,046). Written informed consent was obtained from all individual participants included in the study. All research was performed in accordance with the Declaration of Helsinki.　Clinical information from all patients were obtained based on the medical record. All EC patients underwent upper gastroscopy for pre-treatment assessment, while non-EC controls underwent upper gastroscopy for health check or secondary complete check-up of esophageal or stomach cancer following to barium X-ray examination, or for the complaint of abdominal discomfort. Upper gastroscopy confirmed all non-EC controls were free of esophageal disease including EC. All participants including EC patients and non-EC controls did not have history of severe systemic disease, infection or malignancy in other organ. All participants also did not have history of taking antibiotics for three months before sample collection. Esophagogastroduodenoscopy (EGD) was performed for all patients. To obtain endoscopic esophageal washes, patients were required to swallow a liquid solution (100 ml of water containing 80 mg of dimethylpolysiloxane Gascon: Kissei Pharmaceutical Co., Ltd., Matsumoto, Japan, 1 g of sodium bicarbonate, and 20,000 units of pronase (Pronase MS:Kaken Pharmaceutical Co., Ltd., Tokyo, Japan) before endoscopic examination. After the endoscope was inserted into the esophagus, the endoscopist washed the esophageal wall with a washing solution of 5% Gascon in water. 50 ml of wash solution was applied to the entire esophageal wall, with no exclusive focus on areas that appeared abnormal. Then the washes were immediately aspirated through the suction channel of the endoscope into specimen collection containers (No. 23G0195: Argyle™ Fukuroi, Fukuroi, Japan). The specimen collection container was directly connected to the endoscope modulator, and the washes were vacuumed manually. The samples were immediately　centrifuged and the pellets were frozen at − 80 °C for the molecular analysis. For the EC patients, endoscopic biopsies were also obtained from EC tissues as well as their normal appearing esophageal mucosa. A biopsy specimen was carefully cut into 2 pieces. One was evaluated pathologically and the other was immediately frozen and stored at − 80 °C for the molecular analysis. Genomic DNA was extracted from the pellets or frozen biopsies by the genomic DNA precipitation method. Briefly, biopsy specimen was incubated overnight at 55 C using the Lysis solution containing 25 mM EDTA pH8.0, 2% SDS and proteinase K. Then, the lysate was precipitated and washed by the isopropyl alcohol, 80% ethanol, respectively. The genomic DNA was finally suspended in the TE buffer (pH8.0).

### 16S rRNA gene amplicon sequencing

Using the DNA from biopsy specimen, the 16S rRNA gene was amplified using primers 341F targeting the V3–V4 hypervariable regions (https://jp.illumina.com/content/dam/illumina-marketing/apac/japan/documents/pdf/2014_techsupport_session3.pdf). The 5′end of the primers included the universal sequence of the Illumina adapter. Samples underwent denaturation at 95 °C for 3 min; 35 cycles at 95 °C for 30 s, 55 °C for 40 s, and 72 °C for 40 s; and a final elongation at 72 °C for 5 min. The PCR products were purified and the Nextera XT Index kit (Illumina Inc., San Diego, CA) was added, followed by an additional twelve cycles of PCR. The pooled DNA library was sequenced using a next-generation sequencer MiSeq platform (Illumina Inc.) with MiSeq Reagent Kit version 3 (2 × 300 bp Paired-End Reads, Illumina Inc.). The sequence results were processed using Quantitative Insights into Microbial Ecology (QIIME) pipeline (Ver.2023.2). Primer-trimmed sequences were clustered to amplicon sequence variants using the q2-dada2 plugin and sequences with anonymous bases and chimera were filtered. After quality filtering, Operational taxonomic units (OTUs) were clustered based on 99% sequence similarity with at least 10 identical sequences and assigned against the curated Greengenes v.13.8 reference database at the QIIME web site (http://qiime.org/home_static/dataFiles.html).

Alpha diversity measures were calculated by QIIME. The mean relative abundance (percentage among all reads) in each group was compared at the phylum and genus levels.

### Statistical analysis

Continuous variables between two groups were assessed using the Man Whitney U test.

Correlations of continuous variables between two groups were assessed using the Pearson correlation coefficient. Diagnostic value was evaluated using the receiver operating characteristic (ROC) curve as well as area under curve (AUC), sensitivity and specificity. Association between clinicopathological factors and abundance of specific bacteria were assessed using the univariate and multivariate analyses. An unsupervised hierarchical clustering analysis was used to identify distinct subgroups based on the microbiome status. The *p* value < 0.05 was considered as statistically significant.

### Ethics approval

Approval of the research protocol by an Institutional Review Board: This study was approved by the Institutional Review Board of Fujita Health University (ID: HM18-094) and Kansai medical university (ID: 2,021,046).

### Informed consent

Written informed consent was obtained from all individual participants included in the study.

## Results

### Clinicopathologic characteristics of study cohort

The clinicopathological characteristics of study participants are shown in the Table 1. Age of EC patients was significantly higher than non-EC controls. Patients who had smoking history was also significantly frequent in EC patients than in non-EC controls. Male gender and patients who had alcohol history tended to be frequent in EC patients than in non-EC controls. EC patients consisted of 42 squamous cell carcinomas (SCC) and 3 adenocarcinomas.

**Table 1:** Clinicopathological characteristics of the study cohort.

Variables	EC	Non-EC	*P*
	Patients	Controls	
	(n = 45)	(n = 20)	
Gender: M/F	38/7	12/8	0.053
Age: median (range)	70.0 (46–82)	57.0 (39–77)	0.0001
Smoking history yes/no	38/7	8/9	0.007
Alcohol history yes/no	34/9	9/8	0.059
SCC/Adenocarcinoma	42/3	NA	NA
Stage of cancer (0/I/II/III/IV)	(10/8/6/12/8)	NA	NA

*EC* Esophageal carcinoma, *SCC* Squamous cell carcinoma, *NA* Not applicable.

### Identification of microbiome in endoscopic esophageal washes that discriminate EC patients from non-EC controls

16S rRNA gene amplicon sequencing was performed in endoscopic esophageal washes in patients with 45 primary EC and 20 respective non-EC controls. For the EC patients, the same analysis was also performed in primary EC and their matched normal esophageal biopsies. After quality filtering, a mean of 35,053 sequences per sample (min: 5,008, max: 124,817) were obtained. Read counts of the biopsy (mean:39,190, min: 5,008, max: 124,817) was significantly larger and seemed to have broad range compared to the wash (mean:30,152, min: 13,642, max: 61,613, biopsy vs. wash: *P* = 0.014), while the biopsy sample with minimum filtered depth achieved more than 5000 filtered reads, we included all samples for data analysis.

**Figure 1:**
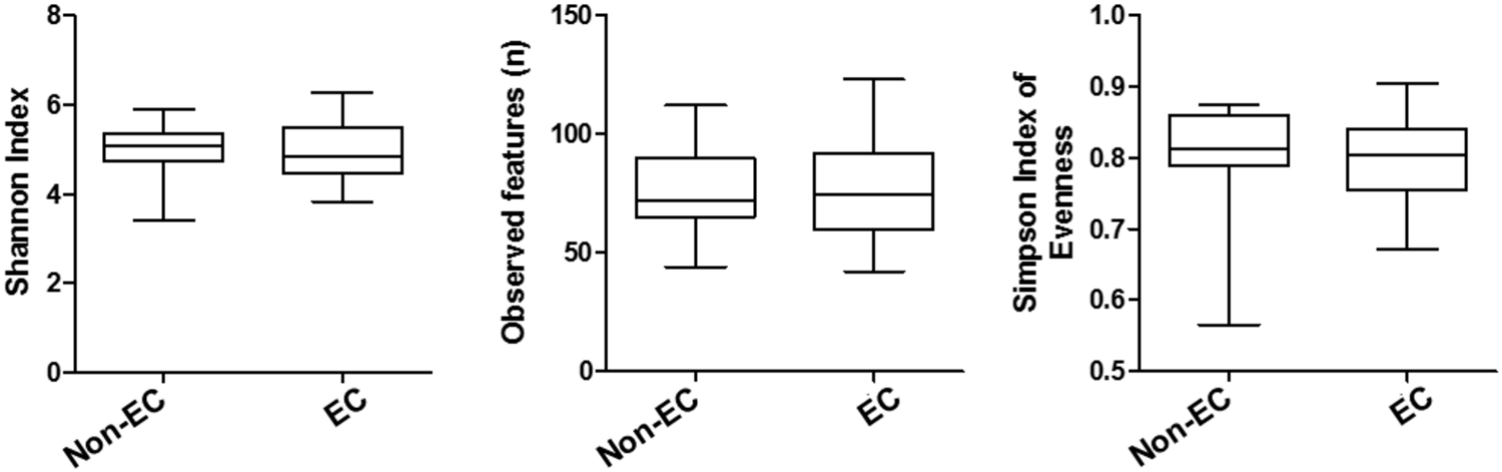
Three indicators of alpha diversity measures, Shannon Index, Observed features and Simpson Index of Evenness among the esophageal washes from EC patients (EC) and non-EC controls (non-EC). Statistical analysis was perfromed using the Mann–Whitney U test.

Initially, we compared three indicators of alpha diversity measures, Shannon Index, Observed features and Simpson Index of Evenness among the esophageal washes from EC patients and non-EC controls. We did not find significant differences of these three indicators of alpha diversity measures among esophageal washes from EC patients and non-EC controls (Fig. 1). When comparing alpha diversity measures among esophageal wash and biopsy samples from EC patients, two of three indicators (Shannon Index and Observed features) were significantly higher in esophageal wash samples compared to that in normal esophageal biopsy, while the Simpson Index of Evenness was also significantly higher in esophageal wash samples compared to that in EC tissues (Fig.S1). On the other hand, we also found that those three indicators of alpha diversity measures in the esophageal wash samples significantly correlated with that in the normal esophageal biopsy, but not the EC tissue biopsy (Fig.S2), suggesting that microbiome in the esophageal wash samples reflect microbiome in the normal esophageal mucosa but not in the EC tissue.

**Figure 2:**
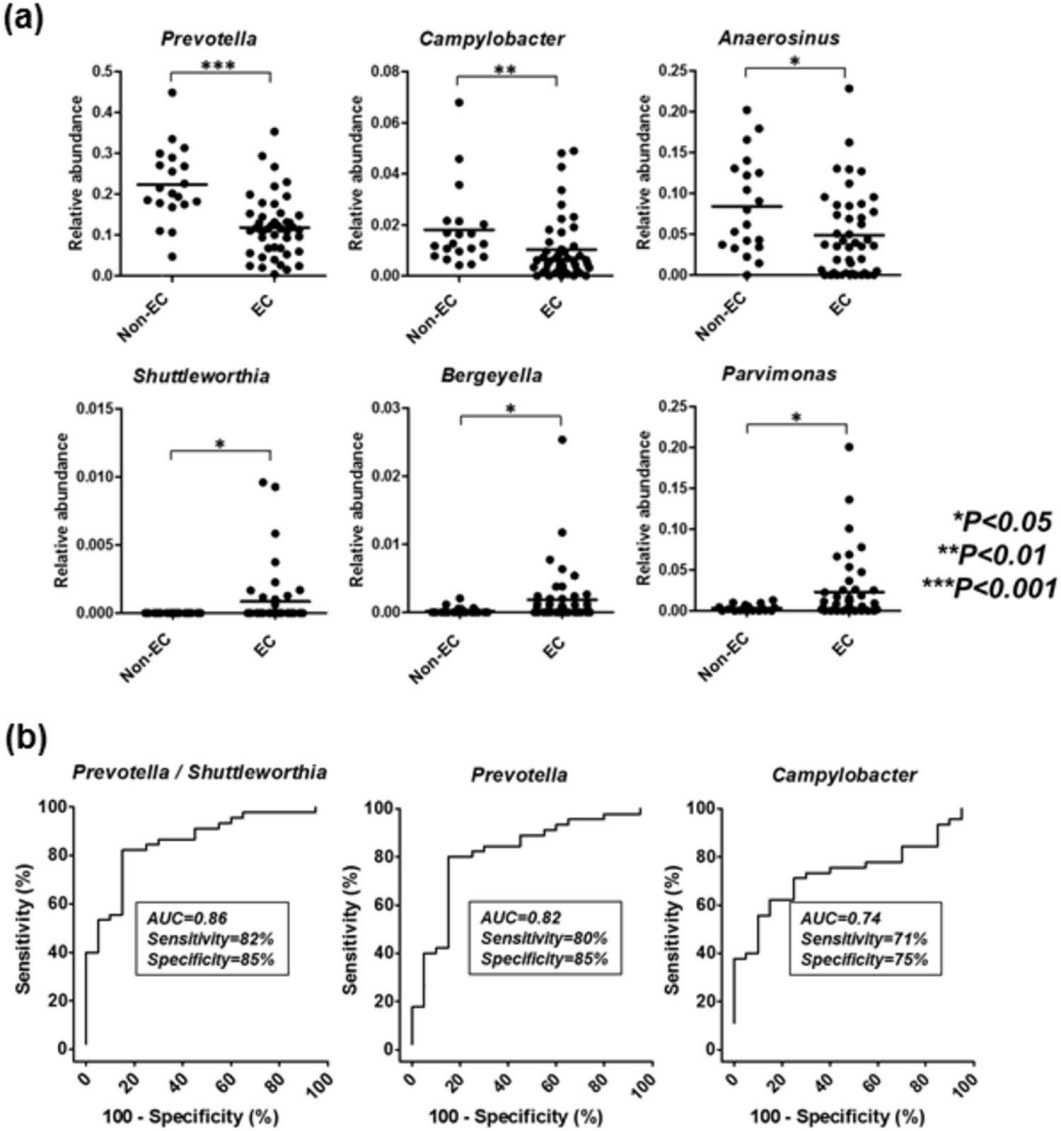
(**a**) Specific bacteria at the genus level in the endoscopic wash samples to discriminate esophageal carcinoma (EC) patients from non-EC controls. Statistical analysis was perfromed using the Mann–Whitney U test. (**b**) Diagnostic value of bacterial taxa in the endoscopic wash samples using single or multi-panels. Each cutoff value was determined using ROC curves.

We then searched for specific bacteria at the genus level to discriminate EC patients from non-EC controls. Comparison of Phyla more than 1% of relative abundance demonstrated significant decrease in *Bacteroidetes* among EC patients than in non-EC controls (19.6% *vs.* 29.5%, *P* < 0.0001), while relative abundances of other phyla did not differ between EC and controls (Fig.S3). At the genus level, we detected 6 discriminative bacterial genera in EC patients (Fig. 2a). Among them, relative abundance of 3 were decreased (*Prevotella*, *P* < 0.001; *Campylobacter*, *P* < 0.01; and *Anaerosinus P* < 0.05) and 3 were increased (*Shuttlewarthia*, *Bergeryella*, and *Parvimonas,* all *P* < 0.05) in esophageal washes from EC patients compared to that in non-EC controls. To further evaluate diagnostic value of above specific genera to discriminate EC patients from non-EC controls, we analyzed the sensitivity and specificity of the above 6 specific genera statistically using single or multi-panels. Each cutoff value was determined using ROC curves (Fig. 2b). The best results were 82% sensitivity and 85% specificity (AUC = 0.86) using relative abundance ratio of *Prevotella* and *Shuttlewarthia.* Relative abundance of *Prevotella* and *Campylobacter* alone demonstrated moderate accuracies for to discriminate EC patients from non-EC controls (*Prevotella,* 80% sensitivity and 85% specificity, AUC = 0.82; *Shuttlewarthia,* 71% sensitivity and 75% specificity, AUC = 0.74). For other specific genera, diagnostic value was limited with the are AUC less than 0.7 (data not shown).

### Clinical factors associated with relative abundance of Prevotella

Since relative abundance of genus *Prevotella* in the esophageal washes best discriminated EC patients from controls as a single panel. We investigated whether abundance of the *Prevotella* would be associated with specific clinicopathological features (Table 2). Univariate analysis demonstrated that, in addition to the occurrence of EC (*P* < 0.0001), smoking habit was also significantly associated with decrease of this genus (*P* = 0.04), but multivariate analysis using the multiple regression model showed only the occurrence of EC was an independent factor associated with decrease of this bacteria (*t* = -3.72, *P* = 0.0005, Table3).

**Table 2:** Association between relative abundance of *Prevotella* and clinicopathological factors.

Variables	Relative abundance (+/−SE)	*P*
Age		
< = 65y (29)	0.16 ± 0.02	Reference
> 65y (36)	0.14 ± 0.02	0.32
Gender		
Female (15)	0.18 ± 0.03	Reference
Male (50)	0.14 ± 0.02	0.54
Smoking history		
**Non smoker (16)**	**0.20 ± 0.03**	**Reference**
**Past or current smoker (46)**	**0.13 ± 0.17**	**0.035**
*Drinking history*		
Non drinker (18)	0.17 ± 0.03	Reference
Past or current drinker (42)	0.14 ± 0.01	0.57
Cancer occurrence		
**Cancer free (20)**	**0.22 ± 0.02**	**Reference**
**Cancer (45)**	**0.12 ± 0.01**	** < 0.0001**

Statistical analysis was performed using the Mann–Whitney U test.

Significant results were expressed as bold characters.

Drinking and smoking history were not determined for 5 and 3 patients, respectively.

**Table 3:** Multivariate analysis using multiple regression model for assessing factors associated with relative abundance of *Prevotella. *Significant values are in bold.

Variables	t	*P*
**Cancer occurrence**	** − 3.72**	**0.0005**
Smoking history	− 1.32	0.19

### Correlation between microbiome in endoscopic esophageal washes and biopsy samples

**Figure 3:**
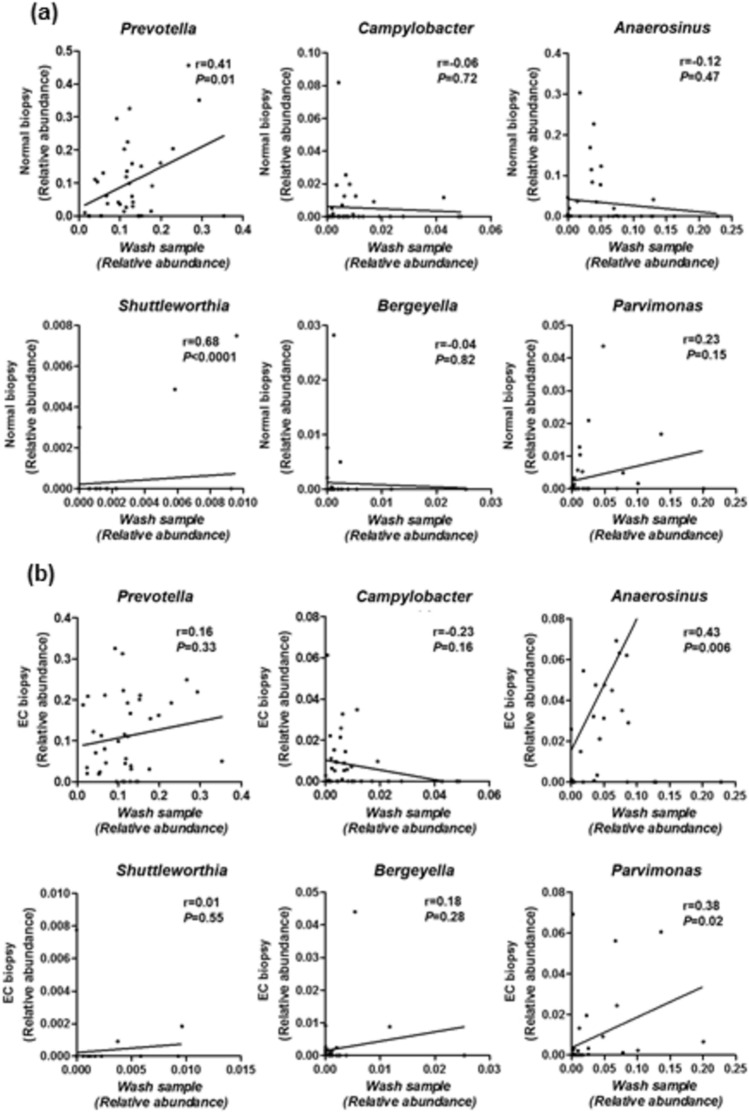
Correlation between microbiome in endoscopic esophageal washes and biopsy samples. (**a**) Correlation between microbiome in endoscopic esophageal washes, biopsy samples from normal esophagus (Normal biopsy). (**b**) Correlation between microbiome in endoscopic esophageal washes, biopsy samples from esophageal carcinoma (EC). Statistical analysis was performed using the Pearson correlation coefficient.

We investigated the correlation between microbiome in endoscopic esophageal washes and that in primary EC and their matched normal esophageal biopsies. Comparison of primary EC and its normal esophageal biopsies identified nine genera with significant change between primary EC and normal esophageal biopsies (Table S1). Among them, five were increased (*Streptobacillus*, *Lachnoanaerobaculum*, *Leptotrichia*, *Peptococcus* and *Moryella*) and four were decreased (*Staphylococcus*, *Acinetobacter*, *Streptococcus* and *Pseudomonas*) in primary EC tissues relative to that in normal esophageal biopsies. However, none of these nine genera overlapped with the 6 discriminative genera shown in Fig. 2. On the other hand, we found that relative abundances of *Prevotella* and *Shuttlewarthia* in the esophageal washes significantly correlated with these in normal esophageal biopsies from EC patients (*P* = 0.01, < 0.0001, respectively, Fig. 3a), while relative abundances of *Anaerosinus* and *Parvimonas* in the esophageal washes significantly correlated with these in primary EC biopsies (*P* = 0.006, 0.02, respectively, Fig. 3b).

Finally, we performed an unsupervised clustering analysis based on microbiome status to see whether overall microbiota composition could identify specific groups of samples (Fig.S4). This analysis included genera more than 1% of relative abundance and the *Fusobacterium* that have previously been linked to the EC^25^. We also included genera with significant differences between endoscopic washes from EC patients and non-EC controls, primary EC and normal esophageal biopsies. Although washes non-EC controls relatively clustered together when compared to washes from EC patients (Fig. S4(a)), distinct cluster was not observed in relation to the primary EC and normal esophageal biopsies (Fig. S4(b)).

## Discussion

The directional association between microbial composition and various digestive cancers suggest interactive role of bacterial microbiota dysbiosis with host immuno-environment while it remains to be tested whether microbial pathogens promote the EC tumorigenesis, which will aid in the management of high-risk patients for a future follow-up surveillance.

In this case–control study, we characterized the microbial communities in EC patients and non-EC controls using endoscopic fluid samples. When comparing alpha diversity measures among esophageal wash and biopsy samples, we found that two of three indicators (Shannon Index and Observed features) were significantly higher in esophageal wash samples compared to that in normal esophageal biopsy, while the Simpson Index of Evenness was also significantly higher in esophageal wash samples compared to that in EC tissues. Although such higher alpha diversity measures shown in wash samples may be due to the contamination of oral bacteria, we also found that those three indicators of alpha diversity measures in the esophageal wash samples significantly correlated with that in the normal esophageal biopsy, but not the EC tissue biopsy, suggesting that microbiome in the esophageal wash samples reflect microbiome in the normal esophageal mucosa but not in the EC tissue. Although we did not observe significant differences in the alfa diversity measures in EC patients and non-EC controls, we identified one and six discriminative taxa at the phylum and genus levels, respectively. ROC analysis of single or multi-panels at the genus level demonstrated good prognostic value of relative abundance ratio of *Prevotella* and *Shuttlewarthia* as well as *Prevotella* alone for discriminating EC patients from non-EC controls (AUC = 0.86, 0.82, respectively). These findings imply a potential utility of bacterial markers as less-invasive and more efficient diagnostic tool for clinical screening of patients who have risk for developing EC. Although histological assessment of endoscopic biopsy is the standard for diagnosis EC, our findings may provide more appropriate clinical implementation combining bacterial markers and the conventional endoscopic examination to predict high risk patients, which would be potentially useful to decide follow-up interval of endoscopic examination. This could potentially decrease the endoscopy related adverse events.

Regarding specific phyla and genera associated with EC, recent study reported the microbial composition in esophageal SCC using esophageal lavage samples^26^. They reported that *Bacteroidetes* and *Fusobacteria* were significantly enriched whereas *Actinobacteria* and *Elusimicrobia* were significantly depressed in esophageal SCC cases at the phylum. At the genus level, a total of 18 genera were significantly enriched in esophageal SCC cases and the relative abundances of 14 genera decreased, respectively^26^. In the present study, there was rather decrease of *Bacteroidetes* among EC patients than in non-EC controls, while other phyla more than 1% of relative abundance did not differ between EC and controls. At the genus level, only one out of 6 discriminative bacterial genera detected in our dataset corresponded to above study (*Shuttlewarthia*). Regarding a decrease of *Prevotella*, a most discriminative genera in our study, opposed results were reported in above and other studies using primary tissue samples^26,28^. In the comparison of microbiome in primary EC and matched normal esophageal biopsies in our data set, we showed five (*Streptobacillus*, *Lachnoanaerobaculum*, *Leptotrichia, Peptococcus* and *Moryella*), and four (*Staphylococcus*, *Acinetobacter*, *Streptococcus and Pseudomonas*) genera were increased, and decreased in primary EC tissues relative to their normal esophageal biopsies, respectively. Among five increased genera in our dataset, the *Leptotrichia* was reported to be enriched in both esophageal SCC and adenocarcinoma tissues^28–30^. On the other hand, *Peptococcus* and *Moryella* are reported as rather decreased in saliva samples in EC patients^31^, while the *Moryella* has been increased in esophageal adenocarcinoma tissues^28^. Regarding four decreased genera in our dataset, *Staphylococcus* and *Streptococcus* were also decreased in esophageal lavage samples from esophageal SCC patients^26^. As shown in above, EC-related microbiome studies have yielded several contradictory results. This inconsistency may be partly due to racial difference and patient’s constitution. For example, the gut microbiome of the Japanese is considerably different from those of other populations and is also highly variable among the same population^32^. Other reason that can potentially explain inconsistent result between our study and recent other study^26^ would be the difference in methodology by which samples were collected. In our study, endoscopic wash fluids were obtained, applying 50 ml of wash solution to the entire esophageal wallwith no exclusive focus on areas that appeared abnormal, while in other study, esophageal lavage samples were collected using 10 ml saline solution^26^, which may not be enough to cover broad area of the esophagus. Since the esophagus is long shaped organ, our methods would potentially reflect bacterial communities in the entire esophagus rather than the focal point compared to other study^26^. Indeed, relative abundances of *Prevotella* and *Shuttlewarthia* in the esophageal washes, both are selected as best discriminative multi-panels, were significantly correlated with these in normal esophageal biopsies from EC patients rather than the primary EC tissues. Since both *Prevotella* and *Shuttlewarthia* were not listed as the genera with significant changes in primary EC tissues relative to their matched normal esophageal biopsies in our dataset, it is reasonable to speculate that microbial communities in the esophageal endoscopic wash fluids reflects resident flora in esophageal mucosa, rather than the bacteria enriched locally in the cancerous tissue. In our data set, biopsy samples had more broad range of sequence reads per sample, while the biopsy sample with minimum filtered depth achieved more than 5000 filtered reads, suggesting that the sequence depth does not affect the quality of our results.

Univariate analysis showed that decrease of *Prevotella,* a most discriminative single panel, was significantly associated with smoking habit, a known lifestyle risk factors for both esophageal SCC and adenocarcinoma^2,33–36^. Multivariate analysis showed only the occurrence of EC was an independent factor associated with decrease of this bacteria. This issue suggests that microbial communities in the esophageal endoscopic wash fluids, reflecting resident flora in the esophagus, are strongly correlate with the occurrence of EC. Decrease of *Prevotella* has been associated with chronic obstructive pulmonary disease (COPD) and its severity^37,38^, a chronic inflammatory lung disease, which is also associated with smoking habit^39^. The result suggests that esophageal resident flora mirror lifestyle and environmental factors that is associated with EC risk. Since exogenous exposures by the known lifestyle and environmental risk factors impact on genotoxic changes in both benign and cancerous tissues^40,41^ and such genotoxic changes may be influenced by host-microbiota interactions^42,43^. Further study will be needed to clarify how our results shown here are relevant in such process.

Present study was from a relatively small number of patients in a few institutions from central Japan. We collected patients consecutively and upper gastroscopy confirmed all non-EC controls were free of esophageal disease including EC. We did not included patients who have history of severe systemic disease, infection or malignancy in other organ, and history of taking antibiotics before sample collection. However, our results from such small samples could not exclude the possibility of selection bias. The study was conducted in a single cultural and geographical setting (Japan), which might also influence the microbiome profiles observed. In our dataset, smoking and alcohol histories, major risk factors of EC were noted but information about other lifestyle factors such as diet, medication use were not available. Since patients with EC included here contained only few cases with adenocarcinoma, we could not evaluate disease associated microbiome according to its histologic difference. In addition, a long-term follow-up was not carried out, and patients with high carcinogenic potential were not subsequently evaluated in non-EC controls. On the other hand, these limitations would be of interest with respect to future EC risk stratification, and further case accumulation is necessary in a prospective manner. Finally, the findings revealed here is a phenotype-microbiome association but not the causality for convergent microbiome dysbiosis in EC. Our finding can be clinically applicable for bacterial markers as less-invasive and more efficient diagnostic tool for clinical screening of EC patients. However, the findings lack evidence for clinical outcomes such as disease progression, recurrence or response to treatment, which would be of interest in the future direction. Further characterization of the mucosal microbiota in the tumor microenvironment using comprehensive approaches will help elucidate host-microbiota interactions underlying carcinogenesis mechanisms.

### Supplementary Information

Supplementary Information.

## Data Availability

All data generated or analyzed during this study are included in this published article and its supplementary information files.
