# Supplementary material for: Microbiome of esophageal endoscopic wash samples is associated with resident flora in the esophagus and incidence of cancer
Source: Sci Rep. 2024 Aug 22;14:19525. doi: 10.1038/s41598-024-67410-1 (PMC11341785; doi:10.1038/s41598-024-67410-1)
Supplement: Supplementary file 1 — Supplementary Information. [file 41598_2024_67410_MOESM1_ESM.docx]

**Supporting information**

| **Supplementary Table 1.** List of genera with significant changes between primary EC and matched | | | |
| --- | --- | --- | --- |
| normal esophageal biopsies | | | |
| Genus | Mean relative abundance | | Mann-Whitney U test (P-value) |
|  | Normal | EC | Normal/EC |
| *Streptobacillus* | 0.001 | 0.003 | 0.0003 |
| *Staphylococcus* | 0.024 | 0.002 | 0.004 |
| *Lachnoanaerobaculum* | 0.002 | 0.006 | 0.008 |
| *Acinetobacter* | 0.005 | 0.000 | 0.01 |
| *Leptotrichia* | 0.006 | 0.028 | 0.01 |
| *Peptococcus* | 0.001 | 0.005 | 0.01 |
| *Moryella* | 0.001 | 0.005 | 0.03 |
| *Streptococcus* | 0.198 | 0.115 | 0.03 |
| *Pseudomonas* | 0.006 | 0.000 | 0.04 |
| Normal, adjacent non-neoplastic esophagus; EC, esophageal carcinoma; | | | |
| Statistical analysis was performed using the Man Whitney U test. | | | |

**
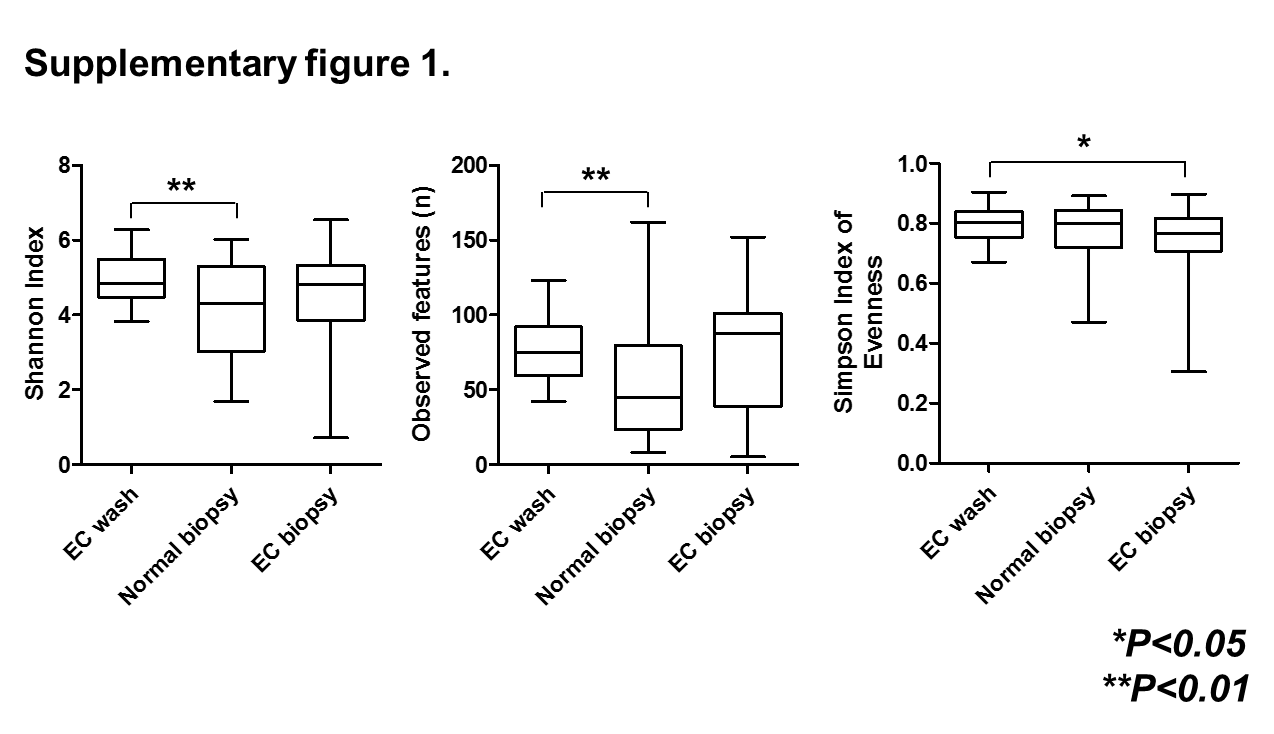
**

**Supplementary figure 1.**

Three indicators of alpha diversity measures, Shannon Index, Observed features and Simpson Index of Evenness among the esophageal washes from EC patients (EC wash), normal esophageal (Normal biopsy) and esophageal carcinoma tissue biopsies (EC biopsies) from esophageal carcinoma patients. Statistical analysis was perfromed using the Mann-Whitney U test.

**
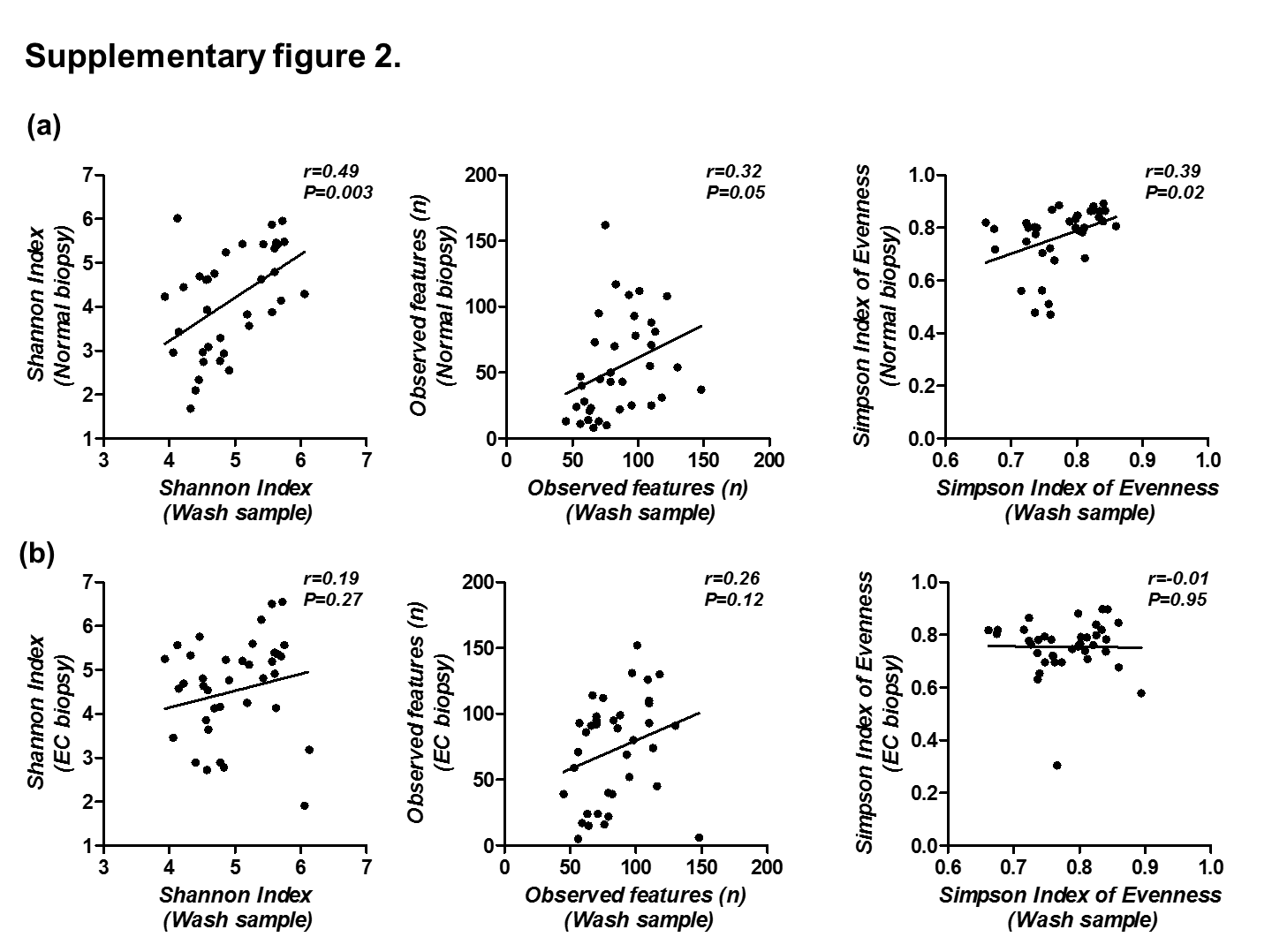
**

**Supplementary figure 2.**

Correlations of three indicators of alpha diversity measures, Shannon Index, Observed features and Simpson Index of Evenness among endoscopic esophageal washes (Wash sample), normal esophageal (Normal biopsy) and esophageal carcinoma tissue biopsies (EC biopsies) from esophageal carcinoma patients. (a) Correlation between endoscopic esophageal washes (Wash sample), biopsy samples from normal esophagus (Normal biopsy). (b) Correlation between microbiome in endoscopic esophageal washes (Wash sample), biopsy samples from esophageal carcinoma (EC). Statistical analysis was performed using the Pearson correlation coefficien

**Supplementary figure 3**

**Supplementary figure 3.**

Comparison of Phyla more than 1% of relative abundance between EC patients and non-EC controls. Statistical analysis was perfromed using the Mann-Whitney U test.

**
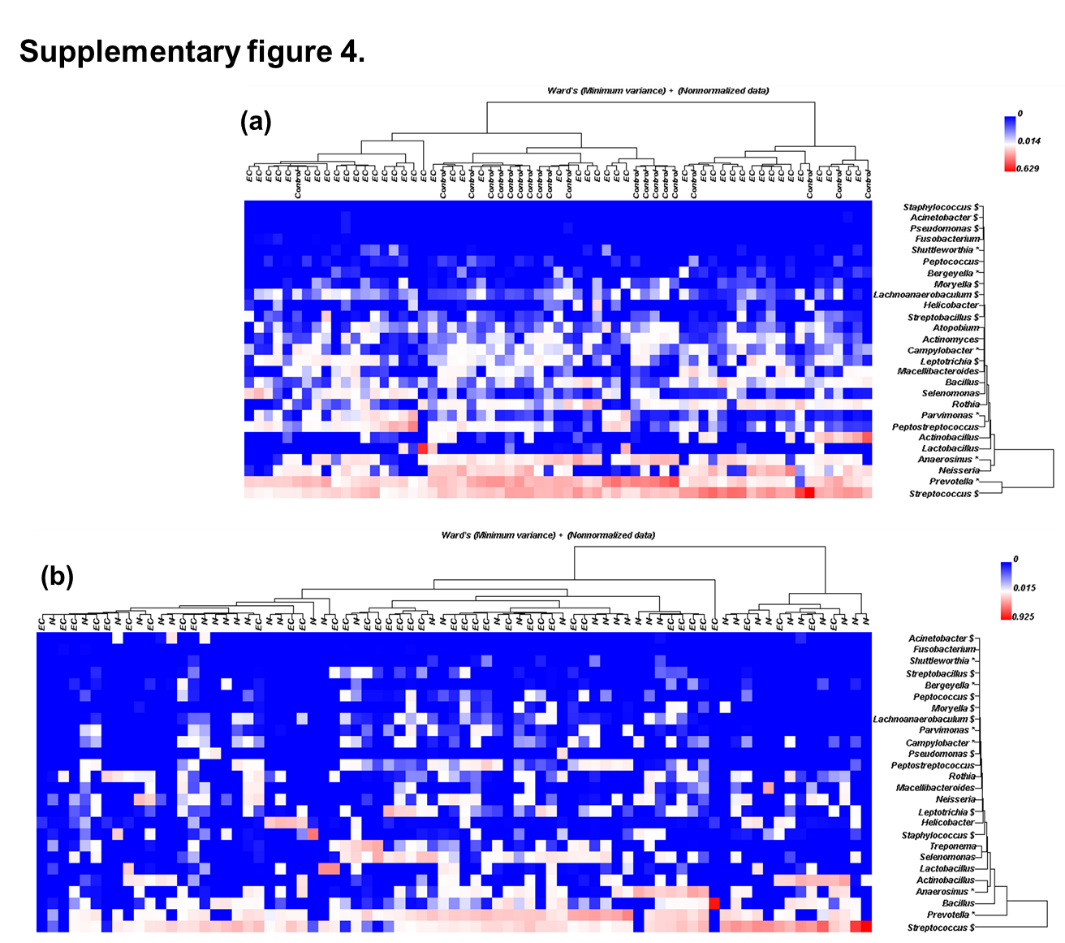
**

**Supplementary figure 4.**

An unsupervised hierarchical clustering analysis to identify distinct subgroups based on the microbiome status. (a) An analysis between endoscopic washes from EC patients and non-EC controls, and (b) primary EC tissues and normal esophageal mucosa. *Genera with significant change among endoscopic washes from EC patients and non-EC controls; $Genera with significant change among primary EC tissues and normal esophageal mucosa;
